# Regulator of telomere elongation helicase 1 gene and its association with malignancy

**DOI:** 10.1002/cnr2.1735

**Published:** 2022-10-17

**Authors:** Mohammad Arian Hassani, Jamshid Murid, Jinsong Yan

**Affiliations:** ^1^ Department of Hematology, Liaoning Medical Center for Hematopoietic Stem Cell Transplantation, Liaoning Key Laboratory of Hematopoietic Stem Cell Transplantation and Translational Medicine, Dalian Key Laboratory of Hematology Second Hospital of Dalian Medical University Dalian China; ^2^ Department of Hematology, Endocrinology and Rheumatology, Ali Abad Teaching Hospital Kabul University of Medical Sciences Jamal mena Kabul Afghanistan; ^3^ Diamond Bay Institute of Hematology Second Hospital of Dalian Medical University Dalian China

**Keywords:** dyskeratosis congenita, glioma, G‐quadruplex, malignancy, *RTEL1*, telomere length

## Abstract

**Background:**

With the progression of next‐generation sequencing technologies, researchers have identified numerous variants of the regulator of telomere elongation helicase 1 (*RTEL1*) gene that are associated with a broad spectrum of phenotypic manifestations, including malignancies. At the molecular level, *RTEL1* is involved in the regulation of the repair, replication, and transcription of deoxyribonucleic acid (DNA) and the maintenance of telomere length. *RTEL1* can act both as a promotor and inhibitor of tumorigenesis. Here, we review the potential mechanisms implicated in the malignant transformation of tissues under conditions of *RTEL1* deficiency or its aberrant overexpression.

**Recent findings:**

A major hemostatic challenge during *RTEL1* dysfunction could arise from its unbalanced activity for unwinding guanine‐rich quadruplex DNA (G4‐DNA) structures. In contrast, *RTEL1* deficiency leads to alterations in telomeric and genome‐wide DNA maintenance mechanisms, ribonucleoprotein metabolism, and the creation of an inflammatory and immune‐deficient microenvironment, all promoting malignancy. Additionally, we hypothesize that functionally similar molecules could act to compensate for the deteriorated functions of *RTEL1*, thereby facilitating the survival of malignant cells. On the contrary, *RTEL1* over‐expression was directed toward G4‐unwinding, by promoting replication fork progression and maintaining intact telomeres, may facilitate malignant transformation and proliferation of various pre‐malignant cellular compartments.

**Conclusions:**

Therefore, restoring the equilibrium of *RTEL1* functions could serve as a therapeutic approach for preventing and treating malignancies.

## 1 INTRODUCTION

Regulator of telomere elongation helicase 1 (*RTEL1*) protein—encoded by the *RTEL1* gene that was initially called the novel helicase‐like gene—maintains telomere integrity and genome stability.^1^ Depending on the cellular context, both; amplification or downregulation of *RTEL1* may contribute to carcinogenesis, so a precise phenotype–genotype, biochemical, and functional analysis of *RTEL1* mutations is required in order to understand the underlying mechanisms of the disease.^2^ Human cells with *RTEL1* mutations exhibit rapid telomere shortening, proliferative exhaustion, increased senescence, and spontaneous apoptosis. Phenotypically, *RTEL1* mutations are known to be associated with a host of genetic diseases and disorders, including dyskeratosis congenita (DC) and its severe variant, Hoyeraal–Hreidarsson syndrome (HHs),^3^, ^4^, ^5^ bone marrow failure (BMF),^3^, ^4^, ^5^, ^6^, ^7^ very early‐onset monogenic inflammatory bowel disease (IBD) and IBD‐like colitis,^5^, ^8^, ^9^ pulmonary fibrosis,^3^, ^4^, ^10^, ^11^, ^12^, ^13^ liver disease^4^, ^14^ and myeloid neoplasms.^7^, ^14^, ^15^, ^16^ Several *RTEL1* mutants are also associated with an increased risk of glioma.^17^, ^18^, ^19^, ^20^, ^21^ Patients with DC are at increased risk of developing squamous cell cancers (SCCs) of the head, neck and anogenital region; myelodysplastic syndrome (MDS); and acute myeloid leukemia (AML).^22^, ^23^ Although a very high incidence of cancer is observed in DC, patients with HHs usually die early because of severe BMF and immunodeficiency.^24^

In patients with HHs, *RTEL1* deficiency is associated with pathological pathways, such as the reduced availability of the 3′ end of the telomeres for elongation by telomerase,^25^ splicing defects, abnormal intracellular trafficking of the small nuclear RNA pre‐U2, and defects in the recycling of ribonucleoproteins in the cytoplasm.^26^ However, the underlying mechanisms of malignant transformation in the presence of *RTEL1* mutations remain undetermined. Furthermore, how both tissue degeneration and malignant transformation occur concomitantly due to the deficiency of a single specific helicase, such as *RTEL1*, needs to be understood. In this review, we focus on the functional alterations of *RTEL1* and their possible association with malignant transformation. Specifically, we examine how malignancy could develop in different cellular compartments under *RTEL1* dysfunction or excess *RTEL1* activity.

## 2 METHODS USED FOR LITERATURE REVIEW

Searches for relevant information were carried out in the PubMed database with no applied filters, using the following terms: *RTEL1*, G‐Quadruplex, mitotic DNA synthesis, (G‐Quadruplex) AND (helicase), (G‐Quadruplex) AND (Werner's syndrome protein), (cancer) AND (telomere maintenance), (alternative lengthening of telomeres) AND (cancer), (inflammation) AND (cancer), (immunodeficiency) AND (cancer), (dyskerin) AND (cancer), (replication fork) AND (speed). The information was extracted from the eligible articles written in English.

## 3 *RTEL1* DEFICIENCY PROMOTES TUMORIGENESIS

### 3.1 Defective G4 structures unwinding during *RTEL1* deficiency causes genomic instability

A study conducted using fluorescence lifetime imaging microscopy reveal that the number of G4 structures (G4s) increased in live cells afflicted by *RTEL1* dysfunction, implicating *RTEL1* in G4‐unwinding.^27^ *RTEL1* resolves telomeric G4s and unwinds telomeric (T)‐loops,^28^ by unwinding the displacement (D)‐loops formed at the base of the T‐loops.^29^ To unwind T‐loops, *RTEL1* is recruited at the telomeres by telomere repeat‐binding factor 2 (TRF2).^30^, ^31^ However, in the absence of *RTEL1*, T‐loops are processed by a group of endonucleases called synthetic lethal of unknown function nucleases (SLX1‐4), which results in the loss of T‐loops with the consequent disturbance of telomere integrity.^28^, ^32^ Loss of the TRF2—*RTEL1* interaction has been observed in patients with HHs.^30^ *RTEL1* interacts with TRF1 which also promotes its recruitment to the telomeres.^3^ A close association has been observed between G4‐DNA and RNA structures in a co‐occurrence called R‐loops. Recently, several independent studies showed that *RTEL1* could regulate G4‐DNA/R‐loops apart from those at telomeric DNA sites. The inability to unwind G4‐DNAs formed in the displaced strand of RNA–DNA hybrids in both; mouse,^33^ and human^34^, ^35^ *RTEL1* depleted cells results in increased R‐loops and elevated transcription‐replication collisions. In the human genome, the helicase function of *RTEL1* promotes the mitotic DNA synthesis (MiDAS) of G4/R‐loop forming loci such as common fragile sites (CFSs) and telomeres that remain under‐replicated during interphase in response to replication stress (RS).^35^ *RTEL1* is recruited to the under‐replicated loci by the SLX4 nuclease, which, in turn, facilitates the recruitment of two other proteins—radiation sensitive 52 (RAD52) and DNA polymerase delta 3, accessory subunit (POLD3)—both of which are necessary for MiDAS.^35^ In this process, the binding of *RTEL1* to SLX4 promotes the recruitment of both enzymes to nascent DNA, and they can be found closely to active RNA polymerase II with the recruitment of Fanconi anemia (FA) complementation group D2 (FANCD2) at RNA polymerase II. Thus, the complex‐forming interaction of SLX4 with *RTEL1* is required for normal RF progression; abolition of this interaction is observed in patients with cancer and HHs, respectively.^36^ Increased R‐loop formation seems to be implicated in genomic instability and cancer development.^33^, ^35^, ^36^, ^37^, ^38^

Also, *RTEL1* seems to counteract rDNA‐destabilizing events by resolving G4s and by maintaining normal RF progression. Similar to the process during *RTEL1* depletion, a significant decline in the copy numbers of specific rDNA and an increased potential for G4‐DNA formation at specific rDNA sites are observed.^39^ Recently, a study showed that the abundance and localization of telomere repeat‐containing RNAs (TERRAs) are influenced by *RTEL1*. Increased levels of TERRA and reduced TERRA‐containing R‐loops at telomeres are observed during *RTEL1* depletion. In this context, the binding of *RTEL1* to existent G4s on TERRA is mediated independently of its helicase domain. Loss of this function of *RTEL1* may also, in some way, contribute to clinical manifestations of DC and HHs.^40^ Human *RTEL1* is also known to unwind (cytosine thymine guanine)_n_/(cytosine adenine guanine)_n_ trinucleotide‐repeat hairpins, thereby blocking the expansion of triplet‐repeats and suppressing triplet‐repeat‐mediated chromosome fragility.^41^

### 3.2 *RTEL1* deficiency results in both; telomeric and genome wide DNA damage which may lead to tumorigenesis

Mouse *RTEL1* is associated with the replisome and avoids replication fork (RF) stalling or collapse,^42^ it's also involved in DNA double‐stranded break (DSB) repair, homologous recombination (HR), and in promoting the efficient elongation of telomeres by telomerase.^43^ Functionally similar analogs of human *RTEL1* in yeast and *Caenorhabditis elegans* (*C. elegans*) act as an anti‐recombinase, eliminating inappropriate recombination events.^44^ *RTEL1* helicase deficiency leads to germ cell mutagenesis. In *C. elegance*, *RTEL1* deficiency results in simple structural variants in the DNA, such as tandem duplications and an increase in base substitutions, frequently appearing in repetitive and G4s‐containing loci.^45^ In mammalian cells, *RTEL1* deficiency results in numerous complex genomic rearrangements, including chromothripsis, end‐to‐end fusions, and tandem duplications with distant intra‐chromosomal insertions as a result of excessive crossover and heterologous recombination. These disastrous events may result in genomic instability and cancer development.^46^ Inherited germline mutations of *RTEL1* together with other factors implicated in DNA damage can result in shortened telomeres. Shortened telomeres, in turn, have been implicated in the pathogenesis of several degenerative disorders along with epithelial and hematological malignancies. Shortened telomeres result in the exposure of chromosome ends as DSBs in DNA, which activates the DNA damage response (DDR) and tumor protein 53, leading to apoptosis/senescence^47^; but also telomere dysfunction‐induced endoreduplication, which was detected in cells of an individual with an *RTEL1* mutation, was proposed to contribute to cancer development.^3^ Fibroblasts of patients with HHs with *RTEL1* deficiency caused telomere aberrations and led to the appearance of interstitial telomeric sequences.^48^ Similar insertions observed in the embryonic fibroblasts of a *RTEL1*‐depleted mouse line and were suggested to result from aberrant recombination between a broken internal chromosomal site and a telomere.^49^ Taken together, these lines of evidence suggest the prominence of genome‐wide DNA damage during *RTEL1* deficiency.^48^

Gross copy number alterations with chromosomal rearrangements, occurring during replicative crisis induced by telomere attrition, are described as the fusion of a telomere with coding genomic loci precipitated by their transcription. The initiation of these events by DDR activation mechanisms, together with an increased expression of the inflammatory elements of senescence‐associated secretory phenotype (SASP), induces the current transcriptome to direct genomic recombination in a small number of cells in such a way that they avoid apoptosis and gain the capacity of clonal evolution, malignant transformation, and metastasis.^50^ In other instances, some *RTEL1* variants may cause excessive 3ˊoverhang erosion, independent of telomere length (TL), which seems to impair cellular proliferation and promote extensive DDR activation. However, the lack of DDR suppression combined with normal TL may predispose cells to genomic instability and myeloid neoplasms, rather than drive pathways of senescence or apoptosis, as it would in cells with extremely short telomeres.^7^

We can conclude that the fate of *RTEL1*‐deficient cells is likely directed toward malignant transformation and is due to defective G4s unwinding, which results in telomeric and genome‐wide DNA damage that lead to complex genomic rearrangements.

### 3.3 *RTEL1* deficiency‐induced inflammation and immunodeficiency are involved in tumorigenesis

Mutations in *RTEL1* are linked to several pulmonary phenotypes, such as idiopathic pulmonary fibrosis (IPF)^10^, ^11^, ^13^ and lung cancer.^51^ In animal models, telomere dysfunction in alveolar stem cells triggers cellular senescence and the recruitment of inflammatory response.^52^ *RTEL1* deficiency has also been implicated in the etiology of connective tissue disease‐associated interstitial lung disease (ILD) with rapid progression.^53^ An analysis of the methylation status of inflammatory cytokine genes in patients with RA, systemic lupus erythematosus, and primary Sjögren's syndrome showed the DNA region for *RTEL1* to be hypo‐methylated, which is taught to serve as an initiator of the autoimmune signaling cascade in these patients.^54^ In the context of telomere biology disorders, hematologic manifestations may occur with pulmonary fibrosis, both of which may be associated with autoimmune disease phenotypes, such as diffuse lymphoplasmacytic infiltrates of lungs, prominent lymphoplasmacytic infiltrates of bone marrow with clusters of plasma cells and eosinophils, Raynaud's phenomenon, psoriasis, and positive antinuclear antibodies. Cytopenia(s) and ILD patterns may concomitantly share etiologies of both telomere biology disorders and autoimmune diseases, such as RA.^55^ Although IPF is characterized by a mixture of cellular proliferation, interstitial inflammation, and fibrosis with unknown etiology,^56^ a variable immune‐deficient status may exist in patients with DC, HHs, or BMF. This frequently leads to recurrent or chronic infections of the respiratory system, which, in turn, cause chronic inflammation, thereby contributing to fibrotic modification of the lungs.^57^ Patients with IPF are at an increased risk of developing lung cancer.^58^, ^59^, ^60^

*RTEL1* mutations cause primary immune deficiency.^4^, ^5^, ^8^, ^9^ *RTEL1* is broadly expressed in proliferating cells, including lymphocytes.^49^ *RTEL1* mutation‐induced natural killer (NK)‐cell deficiency has been identified in several patients with a broad spectrum of clinical manifestations.^4^, ^5^, ^8^, ^9^, ^61^ *RTEL1*‐deficient bone marrow cells exhibit the impaired proliferative potential of the entire bone marrow and cluster‐of‐differentiation 34 (CD34) positive cells in vitro, with the near total disappearance of these cells after prolonged culture. In vivo, differentiation arrest of B‐cells observed during viral infections resulted in a severe reduction of peripheral B‐cell counts, which in turn resulted in hypogammaglobinemia with an impaired antibody response to specific antigens.^4^ *RTEL1*‐deficient T‐cells exhibit increased spontaneous apoptosis in vitro: T‐cells of heterozygous carriers of a mutant allele of *RTEL1* showed significant telomere shortening upon mitogen‐induced long‐term proliferation, whereas homozygous cells die prematurely. Vulnerability of *RTEL1*‐deficient T‐cells to repeated proliferation stimuli triggered, on the one hand, by infections and, on the other, by their primary loss due to BMF and/or premature senescence, leads to T‐cells deficiency.^4^ The coexistence of an immune factor contributing to BMF in patients with mutations in the telomere biology genes, including *RTEL1*, has also been observed through the presence of clones of paroxysmal nocturnal hemoglobinuria cells in some of these patients and also by the fact that some of patients may respond to immune suppressive therapy.^7^ A study examining the genetic overlap between the datasets of primary immune‐deficiencies and inflammatory diseases such as Crohn's disease, ulcerative colitis, very early‐onset monogenic IBD, and multiple sclerosis; has identified several intersecting loci including the one that contains *RTEL1* and proposed that the inactivating variants of these genes may cause immunodeficiency, while variants causing their subtler modulation may contribute to chronic inflammation.^62^

While chronic inflammation may result in carcinogenesis,^63^ several components of the immune system including T‐cells and NK‐cells are implicated in inhibiting tumor development.^64^, ^65^, ^66^, ^67^ Similarly, in patients with FA, inflammation is the significant pathogenic factor determining the clinical phenotype, including cancer.^68^ In addition to fighting chronic inflammation, individuals with FA, even those with mild BMF, and those prior to developing malignancy, are prone to immune dysfunction.^69^

Based on the diverse evidence discussed in this section, we propose that inflammation and immune deficiency induced by *RTEL1* deficiency can promote tumorigenesis.

### 3.4 How do malignant cells survive despite *RTEL1* deficiency?

#### 3.4.1 Telomere maintenance despite *RTEL1* deficiency

Cancer cells may maintain their TLs either by activating telomerase, by alternative lengthening of telomeres (ALT),^70^, ^71^ or by both of them.^72^ In the context of short telomere syndromes, while younger individuals with short telomeres develop aplastic anemia, adults who have relatively longer (but still short) telomeres develop MDS and AML. The relatively longer telomeres seem to potentiate the replicative capacity of hematopoietic stem cells.^73^ Although tumor cells maintain their telomerase activity, telomeres in most of cells are shorter than those in normal cells. Tumor malignancy is possibly contributed by the upregulation of interferon‐stimulated genes of tumor cells with short telomeres.^74^

Telomere loss is facilitated by the telomerase enzyme in the absence of *RTEL1*.^32^ *RTEL1−mouse* cells fail to unwind G4s and T‐loops during replication, leading to the formation of reversed RFs, which are bound by telomerase and trapped in this structure. Then, the SLX1‐4 nuclease complex cleaves the T‐loop, and the partially replicated and truncated sister telomeres are resolved, resulting in telomere loss and telomeric (T)‐circle formation in the end. Conversely, the depletion of the telomerase recruiter tripeptidyl peptidase 1 inhibits telomere loss by blocking the recruitment of telomerase to the telomeres. In the absence of telomerase, the reversed RFs are restarted, and the T‐loops are unwound by the replisome. These experimental findings are consistent with the clinical phenotypes of patients with DC and HHs, in which highly proliferative tissues and stem cells with higher telomerase activity are affected.^32^ Similarly, an experimental study on human cells showed that transient *RTEL1* depletion causes rapid telomere shortening only in those cells with very long telomeres and high levels of telomerase activity; however, the study also suggested that besides the rapid telomere shortening due to T‐loop excision in some cells, the main cause of telomere shortening associated with *RTEL1* dysfunction is the inability of the telomerase to extend the telomeres.^25^ This is consistent with findings that *RTEL1* depletion in some patients cells generates low levels of,^3^, ^7^ or no T‐circles.^4^, ^9^ While *RTEL1* knockout is lethal,^49^ clinical phenotypes caused by *RTEL1* point mutations may be influenced by the location of the mutation within the gene itself, the type of the isoform affected,^4^, ^9^, ^48^ and the associated expression of other genes, such as the telomerase. Although a significant increase in RF stalling or reversal was not observed in a group of such *RTEL1* deficient cells for which they were verified,^48^ RF reversal may maintain replication when DNA secondary structures, oncogene activation, and exogenous obstacles impede RF progression.^75^ Altogether, these findings suggest that the instantaneous damage to telomeres and the whole genome under *RTEL1* deficiency is tolerated by the cells. Despite continuous telomere shortening, these cells will survive for several generations, and it is the ongoing shortening of telomeres over time that may result in clinical phenotypes.^48^ Whether, *RTEL1* deficient malignant cells use telomerase or ALT and what is their relationship with T‐circles formation remains to be determined.

#### 3.4.2 Genome‐wide DNA maintenance despite *RTEL1* deficiency

The expression of several genes involved in the maintenance and progression of the RF may reduce RS and promote tumor progression. MiDAS is known for its capacity for promoting DNA replication in CFS, telomeres, and repetitive sequences during RS.^76^, ^77^ MiDAS occurs at both sites: telomeres that use ALT or telomerase.^78^ HR can mediate break‐induced replication (BIR) of stalled RFs in a RAD51‐dependent manner or RAD51‐independent manner using RAD52‐POLD3.^75^, ^79^ In contrast, *RTEL1* helicase must be present for efficient MiDAS because the RAD52‐ and POLD3‐mediated DNA synthesis depends on the prior R‐loop unwinding activity of *RTEL1*. Following *RTEL1* depletion, the recruitment of RAD52 and POLD3 to the mitotic chromatin is severely decreased, and the recruitment of FANCD2 is increased. The removal of R‐loop structures has been suggested to be an initial requirement for SLX4‐associated nucleases to find their way to the DNA structures required for the initiation of BIR from stalled forks.^35^ In *Arabidopsis thaliana (A. thaliana)*, in the absence of telomerase, the positive role of RAD51 in the HR process in terms of telomere stability depends on the helicase activity of *RTEL1*.^80^ An HR‐deficient status due to *RTEL1* mutations would seem to be a disadvantage for cell survival, as in patients with HR‐deficient pancreatic adenocarcinoma.^81^ Thus, the key question is, how di malignant cells survive despite *RTEL1* deficiency. This raises the possibility that *RTEL1* is partially redundant: there may be other molecule(s) whose functions overlap with those of *RTEL1* and may compensate for *RTEL1* deficiency in surviving cancer cells. Alternatively, there might be pathways that maintain RF progression other than the one that involves *RTEL1*.

In addition to *RTEL1*, other helicases are also known to act on G4s in vivo such as *FANCJ* helicase, Bloom's syndrome helicase (BLM) and Werner's syndrome protein, participating in genomic stability.^1^, ^82^ *FANCJ* resolves G4s and participates in HR repair of DNA DSBs during the S and G2 phases of the cell cycle. FA complementation group D1, FANCD2, FA complementation group M, and FA complementation group S all counteract R‐loop formation. These proteins play a vital role in RF preservation and genomic stability. Clinically, mutations in the FA genes result in cancer predisposition, especially leukemia and SCCs of the head and neck.^83^ *RTEL1* and *FANCJ* work independently and in parallel to repair replication‐associated DNA damage and maintain 45S rDNA. An experimental study on *Arabidopsis* showed that simultaneous mutations of *RTEL1* and *FANCJ b*, a homolog of human *FANCJ*, decreased the rate of root growth and increased the rate of root cell death compared to plants with a mutation in a single gene. This observation suggests that the concurrent activity of both helicases is required for correct replication. *FANCJ b* plays a role in an interstrand crosslink repair pathway in common with the recq‐like helicase 4 (recql4), a functional homolog of the human BLM helicase. *FANCJ b* and *RTEL1* might complement each other during the unwinding of G4s.^84^ A fluorescence lifetime imaging microscopic study indicated that *FANCJ* alone (in *RTEL1* depleted cells) is less effective in unwinding G4s than *RTEL1* alone (in *FANCJ* depleted cells), but its role cannot be ignored.^27^ Furthermore, the expression of *FANCJ* was strongly induced in *A. thaliana* with *RTEL1* mutation, and *RTEL1* and recql4 acted synergistically in the growth of the wild‐type plant. Interestingly, *RTEL1*‐deficient *A. thaliana* also exhibited increased resistance to hydroxyurea.^85^ Also, its demonstrated that FANCD2 supports MiDAS in parallel with RAD52 in cancer cell lines.^86^ In an independent study, an interaction between *RTEL1* and POLE was observed in both metazoans and mammalians. This interaction is required for the firm synchronization between origin activation and fork elongation during replication, which is necessary for the maintenance of genome stability.^87^ The combined loss of POLE4 (an accessory subunit of POLE) and *RTEL1* results in the termination of DNA replication, accumulation of HR intermediates, genomic instability, and embryonic lethality. In *C. elegans*, in *RTEL1* and *Pole4* double mutants, the Rad51 and replication protein A (RPA) foci accumulate, and replication fails. *RTEL1* and *Pole4* double knockout mice exhibit fork asymmetry and defective origin activation.^87^ An interaction was also observed between *RTEL1* and Poldip3, a protein implicated in DNA synthesis and messenger RNA (mRNA) trafficking; a reduction in the levels of either *RTEL1* or Poldip3 alone reciprocally reduced the accumulation of the other one in the chromatin. The deficiency of both proteins induced the accumulation of recombination intermediates and an increase in nuclear RAD51.^34^ *RTEL1* interacts with RPA, a single‐stranded DNA binding protein that stimulates helicase‐catalyzed DNA unwinding. *RTEL1* requires RPA for the efficient unwinding of T‐loop duplexes in vivo.^88^ In *A. thaliana*, the absence of RPA2, subunit A (RPA2A) induces telomere shortening; however, this telomere shortening is completely abolished in plants lacking *RTEL1*. In fact, in *rpa2a* and *RTEL1* double mutants, TL restoration is induced due to the activation of an HR‐mediated ALT mechanism for telomere replication, which is hypothesized to be inhibited in the presence of *RTEL1*.^89^

**FIGURE 1:**
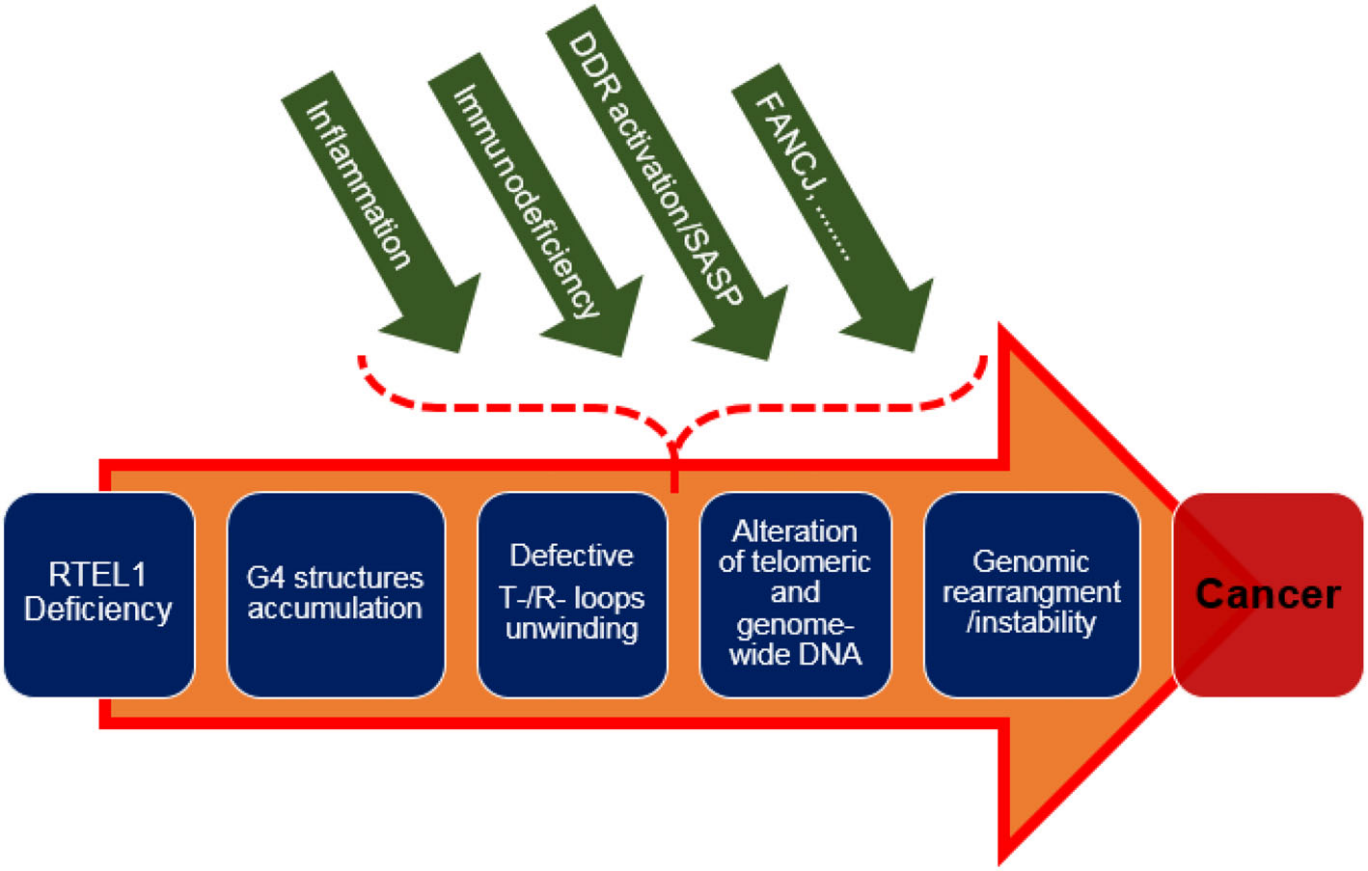
A short illustration of malignant transformation of *RTEL1* deficient cells. Several intrinsic and extrinsic probable facilitator factors are mentioned. DDR, DNA damage response; *FANCJ*, Fanconi anemia complementation group J; G4, guanine quadruplex; SASP, senescence‐associated secretory phenotype.

In this section, we reviewed experimental and clinical evidence regarding the role of different molecules—*FANCJ*, POLE4, and RPA—in particular, in RF maintenance and their functional relationship to *RTEL1*. Based on this collective evidence, it seems probable that, with their function kept intact, these molecules may somehow compensate for the deficiency of *RTEL1* caused by point mutations. This compensation can occur in such a way that *RTEL1* deficient malignant cells may maintain their survival and proliferative capacities. We summarized the involvement of different factors leading to carcinogenesis during *RTEL1* deficiency in Figure 1.

## 4 *RTEL1* OVER‐EXPRESSION/ACTIVITY PROMOTES TUMORIGENESIS

In mice models, *RTEL1* over‐expression resulted in the development of hepatocellular tumors. *RTEL1* could support cell growth due to its potential participation in wingless‐related integration site/β‐catenin signaling.^90^ Several studies have demonstrated the relationship between *RTEL1* variants and the inherited susceptibility to glioma,^17^, ^18^, ^19^, ^20^, ^21^ specific to,^19^ or regardless of its histological subtypes.^18^ Single nucleotide polymorphisms (SNPs) of *RTEL1* associated with glioma are mostly found in non‐coding (intronic) regions of the gene. Some SNPs of *RTEL1* may alter its expression level in the brain tissues.^18^ The genomic 20q13.33 region including *RTEL1* was found to be amplified in nearly 30% of gliomas with copy number change correlating with *RTEL1* expression.^17^ The 20q13.33 genomic region including *RTEL1* may mediate its risk effect in gliomagenesis by regulating the transcriptome through its multiple alternatively spliced transcripts such as exome skipping. SNPs associated with spliced RNA are mostly found within the binding sites for RNA‐binding proteins (RBPs), which may alter the ability of RBPs to bind and interact with pre‐mRNA and other RBPs within a spliceosome.^91^ A functional SNP on the 20q13.33 region in linkage disequilibrium with another SNP mapped to intron 14 of *RTEL1* affects the activity of an enhancer on 20q13.33 that leads to modulated expression of multiple genes implicated in glioma risks, including *RTEL1*. *RTEL1* expression is modulated by this SNP in isocitrate dehydrogenase I wild‐type glioma and during early brain development but not in normal adult brain tissues. Disruption of the region containing this SNP reduced *RTEL1* expression. Because *RTEL1* maintains genomic stability by suppressing HR, it may be more active during gliomagenesis and/or during early brain development.^92^ Another study suggested that over‐expression of *RTEL1* overcomes the tumor‐suppressive effects of microRNA 4530 (miRNA‐4530) in human gliomas, and inversely, miRNA‐4530 over‐expression inhibits the malignant biological behaviors of human glioma cells. These observations indicate that *RTEL1* functions as an oncogene or a tumor suppressor depending on the cellular context.^93^ Furthermore, *RTEL1* is highly expressed in adrenocortical carcinoma and is associated with shorter overall and progression‐free survival.^94^

### 4.1 How does *RTEL1* over‐expression/activity promote tumorigeneses?

The potential explanation for *RTEL1* acting as a tumor promoter hypothetically comes from understanding its functions at the molecular level in several directions.

First, MiDAS is a useful pathway to prevent genomic instability; however, it is also a protector of cancer cells during RS.^76^, ^77^, ^95^ MiDAS inhibition specifically by targeting RAD52 may serve as a therapeutic strategy in the elimination of cancer cells.^96^, ^97^ MiDAS is a *RTEL1*‐dependent process because of the capability of *RTEL1* for resolving G4s‐associated R‐loops at CFSs and telomeres.^35^ G4s have an equilibrated folded‐unfolded status; G4 ligands change the unfolded conformation to the folded one by stabilizing G4s, which may inhibit several biological processes including replication, transcription, and translation. Telomestatin is a known G4 stabilizer and its analogs cause growth inhibition of glioma stem cells in vitro and vivo.^98^ Taken together, this evidence suggests that in some instances, the unwinding of G4s facilitated by *RTEL1* leads to a survival advantage of malignant compartments.

Second, both; stalled RFs or excessive RF speed with reduced stalled forks and consecutive DDR activation may negatively affect cellular viability, the RS and genomic instability resulting from excessive RF speed is not ignorable.^99^ *RTEL1* is recognized for its role in facilitating RF progression.^34^, ^42^, ^87^ Although to the best of our knowledge, no specific reports have detailed the role of *RTEL1* in accelerating RF progression to unusual levels, excess *RTEL1* activity may somehow contribute to the acceleration of RF progression with its undesirable consequences such as genomic instability with cancer promotion.

Third, long TL seems to be associated with an increased risk of a subset of human cancers. The manner in which each SNP affects the TL is allele‐dependent with its consequences.^100^ As an example, the short telomere allele of a certain SNP near the *RTEL1* gene is associated with IPF, and the long telomere allele of the same SNP is associated with lung adenocarcinoma.^101^ Similarly, another study indicated that genetically longer leukocyte TL is associated with *increased* glioma risk.^102^ In individuals with constitutively long telomeres, the cancer is postulated to be originally generated in two stages of mutations. In the initial stage, multipotent stem cells replicating during any period of life accumulate mutations, resulting in clones that have advantages for replication or survival over the surrounding cells lacking these mutations. In the second stage, these previously mutated clones encounter a series of driver mutations that provoke positive selection at the clonal level, more replications of the expanding clones predispose the cells for additional mutations, and all these changes together ultimately lead toward the malignant transformation in a specific tissue where the clones are evolved. Here, in the second stage, the relatively longer telomeres become an appropriate back‐up to support the repetitive replicative cycles of expanding cells.^103^ *RTEL1* has been implicated in preventing telomere fragility and loss through various mechanisms, including the removal of telomeric DNA secondary structures,^28^ the elongation of the single‐stranded telomeric G‐overhang by telomerase,^25^ and the compensation for telomere loss in the absence of telomerase.^80^

**FIGURE 2:**
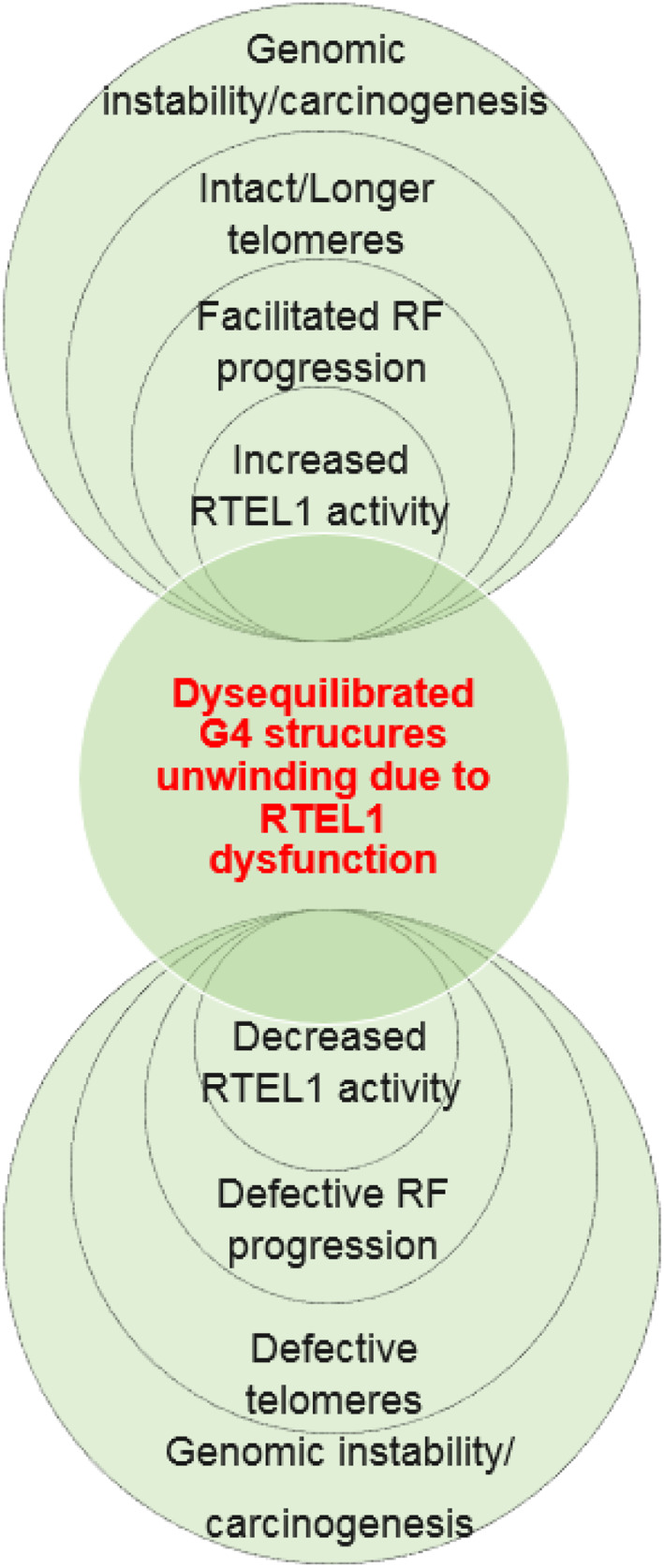
Common and progressive relationships between the potential factors leading to genomic instability due to dysregulated *RTEL1* activity in context of G4 structures unwinding in opposite directions. G4, guanine quadruplex; RF, replication fork.

With the evidence discussed in this section regarding the roles of *RTEL1* in telomere maintenance and the beneficial role of intact telomeres in maintaining the survival capacity of cancer cells, it seems probable that *RTEL1* may act through these mechanisms to facilitate carcinogenesis. The development of genomic instability during *RTEL1* dysfunction is illustrated in Figure 2.

## 5 DISCUSSION

The specific prodromal mechanisms of tumorigenesis of previously normal tissues are now illustrated in a considerable level of detail.^104^ Here, we discussed the probable causative mechanisms of tumorigenesis in patients with a known degenerative disorder such as DC or its severe variant, HHs, characterized by very short telomeres. Mutations in several telomere biology genes including *RTEL1* have been identified and found to be responsible for the clinical manifestations in most cases.^24^ In addition to the role of *RTEL1* in telomere maintenance, non‐telomeric defects caused by *RTEL1* mutations may contribute to the HHs‐specific manifestations.^24^, ^26^ Furthermore, the heterogeneity of clinical manifestations might be related to the tissue‐dependent expression of various *RTEL1* isoforms with their specific functionality.^4^, ^9^

While *RTEL1* deficiency results in the degeneration of various cellular compartments,^4^, ^6^, ^105^ the mutation is also associated with an increased risk of cancer development.^7^, ^14^, ^15^, ^16^ Therefore, for the first time in this review, we intended to explain how malignancy may occur and progress in individuals with *RTEL1* deficiency who already suffer from a degenerative disease, such as DC or other phenotypes, where the replicative capacity of various cellular compartments is terminated due to *RTEL1* dysfunction. *RTEL1* deficiency raises conflicts between the replication and transcription machinery due to defective G4s unwinding,^33^, ^34^, ^35^, ^36^ causes spontaneous DNA damage, short telomeres, telomeric aberrations, anaphase bridges,^6^ and complex chromosomal rearrangements, such as chromothripsis, all of which may lead to genomic instability and cancer development.^46^ Several helicases are already known that act on structures similar to those on which *RTEL1* acts,^1^, ^82^ and helicases/molecules such as *FANCJ*,^84^ and POLE4,^87^ are known to show synergistic activity with *RTEL1*. It raises the questions, if there are other helicases/molecules that participate in compensating for some of the lost *RTEL1* functions and promote the survival of *RTEL1*‐deficient malignant cells which has not been proven yet to the best of our knowledge and should be illustrated further, and whether there might be other DNA maintenance pathways than those that involves *RTEL1*, as in *rpa2a* mutated *A. thaliana* with telomere replication defects, where the telomere shortening was abolished by HR during *RTEL1* deficiency.^89^ It is vital to gain a comprehensive understanding of the mechanisms underlying the replicative ability in *RTEL1*‐deficient malignant cells. Not least important is to learn if crosstalk exists between different helicases, such that the deactivation of one helicase would be compensated by other helicases. The hope is that such insights will positively change the therapeutic approach to malignancies arising from the deficiency of *RTEL1* or other specific helicases. Also, it is worth mentioning that some of *RTEL1* variants seems to be protective in certain pathological phenotypes.^106^, ^107^ Whether the functional mechanisms standing for such pathogenic and/or protective effects of *RTEL1* are the same, so if their amplification and/or down regulation may induce and/or prevent from a certain clinical phenotype or not, and what other factors are implicated in such circumstances, needs to be evaluated further.

Previous studies have indicated that chronic inflammation may play a role in the development and progression of cancer.^108^, ^109^, ^110^ Furthermore, immunodeficiency, whether congenital or acquired, is associated with an increased risk of malignancy.^111^ Similarly, the presence of age‐related chronic inflammation and immunosenescence may significantly affect the development of malignancies in frail individuals.^112^ In contrast, certain anti‐inflammatory drugs^113^, ^114^ and immunotherapies^115^; have been proven to be effective in treating malignancies. Thus, we hypothesize that the presence of an immune‐deficient and/or an inflammatory microenvironment in patients suffering from *RTEL1* deficiency may act as a facilitator of tumorigeneses and could be considered not only as an etio‐pathogenic factor but also as a therapeutic target in such patients.

Furthermore, it will be interesting to know if *RTEL1* dysfunction results in direct translational disturbances and if they contribute to tumorigenesis. A sign to search in such a direction would come from the findings from another gene called dyskerin pseudouridine synthase 1, which encodes a protein called dyskerin, which has been implicated in both telomere maintenance and translational processes. DC caused by mutations in this gene shares phenotypic characteristics with DC caused by mutations in any of the other genes associated with telomerase function or telomere integrity. Dyskerin dysfunction may contribute to the increased susceptibility of patients to cancer development.^116^, ^117^

**FIGURE 3:**
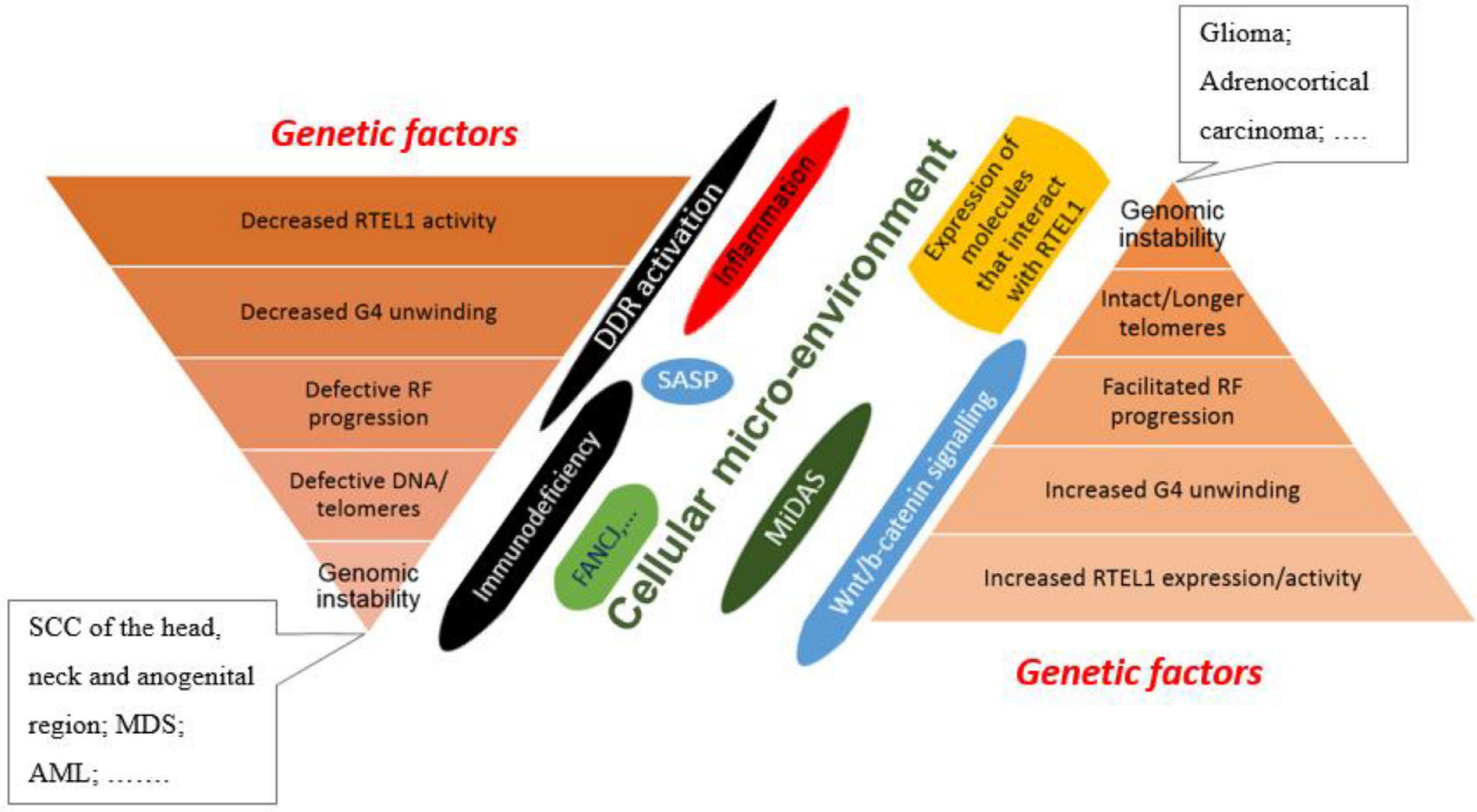
Both; decreased or increased *RTEL1* activity together with several facilitating factors may finally result in genomic instability and carcinogenesis. AML, acute myeloid leukemia; DDR, DNA damage response; *FANCJ*, Fanconi anemia complementation group J; G4, guanine quadruplex; MDS, myelodysplastic syndromes; MiDAS, mitotic DNA synthesis; RF, replication fork; SASP, senescence‐associated secretory phenotype; SCC, squamous cell cancer; Wnt/b, wingless‐related integration site/beta.

Equally important is to understand the pro‐oncogenic role of *RTEL1* that could arise from its over expression and its upregulated activity, which manifests in excessive G4s unwinding and maintaining a higher‐than‐normal speed of RF progression. Thus, targeted interference in the aberrant functions to maintain adequate TL for repetitive cellular divisions could be the key to the development of novel therapeutic strategies for various malignancies. Up/down regulation of *RTEL1* and its association with malignancy is summarized in Figure 3.

## 6 CONCLUSION

From this review, we conclude that tumorigenesis due to both *RTEL1* deficiency and *RTEL1* over‐expression/activation mainly depends on *RTEL1*'s activity in the unwinding of G4s and maintaining RF progression. This, in turn, points to the complex roles that G4s play depending on their stability in facilitating or inhibiting several vital processes of the cell, such as replication, transcription, and translation. It seems that during normal physiological conditions *RTEL1*'s unwinding of G4s and the stability of G4s are in equilibrium. A shift of this equilibrium toward either excessive G4 winding or unwinding would result in genomic instability. Defective unwinding of G4s under the condition of *RTEL1* deficiency stalls the RF, which is a survival disadvantage but also a source of RS that may lead to genomic instability. The concomitant presence of a defective immune system and inflammation in *RTEL1* deficiency may provide the perfect microenvironment for the development of malignancies. Point mutations may damage a certain region of *RTEL1* leading to a loss of one of its specific functions, while its other functions could be preserved and the interacting network of various of molecules functionally similar to *RTEL1* could facilitate the survival of *RTEL1*‐deficient malignant cells. How cellular growth is promoted in malignant cells while *RTEL1* deficiency is present is a key issue that requires further investigation. Similarly, which levels of *RTEL1*'s helicase activity and G4s stability are required for normal cellular growth and how the other helicases participate in compensating for *RTEL1*'s deteriorated functions are other important questions that deserve future investigation. Finally, at the most fundamental level, the deeper understanding of interplay of *RTEL1* with other helicases or other biologically functional molecules is warranted.

## AUTHOR CONTRIBUTIONS

**Mohammad arian Hassani:** Conceptualization (lead); data curation (lead); formal analysis (lead); funding acquisition (supporting); investigation (lead); methodology (lead); project administration (lead); resources (equal); software (lead); supervision (lead); validation (lead); visualization (lead); writing – original draft (lead); writing – review and editing (lead). **Jamshid Murid:** Conceptualization (equal); data curation (equal); formal analysis (equal); funding acquisition (supporting); investigation (supporting); methodology (equal); project administration (equal); resources (supporting); software (supporting); supervision (equal); validation (equal); visualization (equal); writing – original draft (lead); writing – review and editing (equal). **Jinsong Yan:** Conceptualization (lead); data curation (equal); formal analysis (equal); funding acquisition (lead); investigation (equal); methodology (lead); project administration (lead); resources (lead); software (equal); supervision (lead); validation (lead); visualization (lead); writing – original draft (equal); writing – review and editing (equal).

## FUNDING INFORMATION

This work was supported by National Natural Science Foundation of China Grants 81570124, Science and Technology Innovation Leading Talent Program of Liaoning Province (XLYC1902036), Basic Research on the Application of Dalian Innovation Fund (2019J12SN56), Key R & D projects in Liaoning Province (2019JH8/10300027), Key Project of the Educational Department of Liaoning Province (LZ2020003).

## CONFLICT OF INTEREST

The authors declare no conflicts of interest.

## ETHICS STATEMENT

Not applicable.

## Data Availability

My article is a book review.
